# Neurological Complications in Pediatric Takayasu Arteritis: Insights from a Single-Center Experience

**DOI:** 10.3390/jcm15166333

**Published:** 2026-08-16

**Authors:** Esma Sengenc, Sezgin Sahin, Mehmet Yildiz, Huseyin Kilic, Kenan Barut, Serhat Guler, Nergis Akay, Serdar Arslan, Ozgur Kasapcopur, Sema Saltik

**Affiliations:** 1 Department of Pediatric Neurology, Cerrahpaşa Faculty of Medicine, Istanbul University–Cerrahpaşa, 34098 Istanbul, Turkey; 2 Department of Pediatric Rheumatology, Cerrahpaşa Faculty of Medicine, Istanbul University–Cerrahpaşa, 34098 Istanbul, Turkey; 3 Division of Neuroradiology, Department of Radiology, Cerrahpaşa Faculty of Medicine, Istanbul University–Cerrahpaşa, 34098 Istanbul, Turkey

**Keywords:** Takayasu arteritis, pediatric, neurological involvement, stroke, neuroimaging

## Abstract

**Background/Objectives:** Pediatric Takayasu arteritis (PTA) is a rare large-vessel vasculitis with limited data regarding neurological involvement. This study aimed to evaluate neurological manifestations and associated neuroimaging findings in a cohort of children with PTA. **Methods:** In this retrospective observational study, 22 patients diagnosed with PTA and followed at a tertiary center between January 2000 and April 2025 were included. Neurological symptoms, examination findings, and neuroimaging data were reviewed. Angiographic involvement was classified according to the Numano classification. **Results:** Neurological involvement was identified in 5 patients (22.7%), including ischemic stroke (*n* = 3) and hypertensive encephalopathy (*n* = 2). Neurological symptoms were present in 15 patients (68.2%), with headache being the most common manifestation (54%). Other symptoms included dizziness, paresthesia, visual disturbances, syncope, and seizures. Neuroimaging revealed ischemic lesions in a subset of patients, as well as nonspecific white matter changes. Angiographic evaluation showed a predominance of extensive disease patterns, particularly Type V and Type IV involvement. **Conclusions:** Neurological manifestations in pediatric Takayasu arteritis are common but often nonspecific. However, severe complications such as stroke and hypertensive encephalopathy may occur and can be the initial presentation. Careful neurological evaluation and the use of neuroimaging are essential for early detection and appropriate management.

## 1. Introduction

Takayasu arteritis (TA) is a rare, chronic, granulomatous inflammatory large-vessel vasculitis that primarily involves the aorta and its major branches [1,2]. The disease predominantly affects young women of childbearing age and only rarely begins in childhood [3,4]. Although pediatric Takayasu arteritis (PTA) is the most common form of large-vessel vasculitis in children, epidemiological data remain limited and demonstrate considerable geographic variability [5,6].

In PTA, persistent vascular inflammation leads to stenosis, occlusion, or aneurysmal dilatation of major arteries, ultimately resulting in end-organ ischemia. Common clinical findings include systemic symptoms such as fever, malaise, and weight loss, as well as vascular signs including diminished pulses, blood pressure discrepancies, and arterial bruits. Hypertension, most frequently secondary to renal artery involvement, is a common presenting feature in affected children. Due to its progressive course and the risk of potentially life-threatening complications, PTA is associated with a substantial disease burden [5,6].

Recent reviews emphasize the importance of early diagnosis and comprehensive evaluation in PTA because of its widespread and multisystemic involvement [7,8,9].

Neurological involvement in Takayasu arteritis may occur in the form of cerebral ischemia secondary to stenotic involvement of the carotid and vertebral arteries or as hypertensive encephalopathy related to uncontrolled hypertension. Reported clinical manifestations include headache, dizziness, syncope, seizures, transient ischemic attacks (TIA), and ischemic stroke [10,11,12].

This condition, which represents an important cause of morbidity, may remain clinically silent during the early stages of the disease in children. In this context, neuroimaging modalities—particularly magnetic resonance imaging (MRI) and magnetic resonance angiography (MRA)—play a critical role in detecting subclinical central nervous system abnormalities and vascular pathology, thereby facilitating earlier diagnosis and timely therapeutic intervention [13,14].

Takayasu arteritis is angiographically classified according to patterns of arterial involvement. The most widely used system, the Numano classification, categorizes the disease into six types based on the angiographic distribution [15]. This classification guides clinical assessment and therapeutic decision-making. Data regarding the prevalence, clinical spectrum, and neuroimaging characteristics of neurological involvement remain limited, as the available evidence largely derives from small case series and single-center studies [14,16].

This retrospective study was planned to investigate neurological manifestations in a cohort of patients with PTA, to describe the neuroimaging findings associated with the clinical spectrum, and to examine the possible relationship between neurological manifestations and angiographic involvement patterns according to the Numano classification.

## 2. Materials and Methods

### 2.1. Study Design and Population

In this retrospective observational study, medical records of patients diagnosed with PTA and followed at a tertiary pediatric rheumatology center between January 2000 and April 2025 were reviewed. A total of 22 patients with PTA were included in the study. Although neurological examinations were performed during routine follow-up in the rheumatology clinic, the follow-up protocol required that all patients undergo detailed neurological evaluation in the pediatric neurology department at diagnosis, during annual follow-up visits, and when neurological findings were present.

Neurological evaluation included a comprehensive neurological examination as well as cranial MRI, MRA, and electroencephalography (EEG) when clinically indicated.

### 2.2. Inclusion Criteria

All patients were younger than 18 years of age at the time of diagnosis and fulfilled the EULAR/PRINTO/PReS classification criteria for childhood Takayasu arteritis. These criteria require evidence of typical arterial involvement on angiography or cross-sectional imaging, plus at least one of the following: pulse deficit or claudication, blood pressure discrepancy > 10 mmHg, arterial bruit, hypertension, or elevated acute-phase reactants [17]. In the present cohort, vascular involvement was assessed by magnetic resonance angiography (MRA).

### 2.3. Exclusion Criteria

Patients with insufficient medical records or imaging data for adequate evaluation were excluded from the study.

### 2.4. Neurological Assessment and Data Collection

Neurological symptoms, examination findings, and follow-up data were retrospectively collected from medical records. Neuroimaging was re-reviewed by the same neuroradiologist. All evaluated EEG recordings consisted of at least 1-h awake and sleep EEG studies. Final neurological status was assessed based on available clinical follow-up.

Neurological involvement was defined as the presence of neurological symptoms accompanied by corresponding clinical findings, or abnormalities on MRI or EEG considered to be related to Takayasu arteritis (TA). Ischemic stroke was diagnosed based on acute neurological deficits with corresponding diffusion restriction on MRI. Hypertensive encephalopathy was diagnosed in the setting of severe uncontrolled hypertension accompanied by acute neurological symptoms, with or without posterior reversible encephalopathy syndrome (PRES) pattern on MRI. Neurological findings that could not be definitively attributed to Takayasu arteritis—such as those potentially related to procedural complications or comorbid conditions—were not classified as neurological involvement.

Nonspecific neurological complaints reported by the patients were also recorded as neurological symptoms.

### 2.5. Angiographic Classification

Patients were categorized according to the angiographic classification system proposed by Numano et al. [15]: Type I, involvement of the aortic arch branches only; Type IIa, involvement of the ascending aorta, aortic arch, and its branches; Type IIb, involvement of the ascending aorta, aortic arch, its branches, and the descending thoracic aorta; Type III, involvement of the descending thoracic aorta, abdominal aorta, and/or renal arteries; Type IV, involvement of the abdominal aorta and/or renal arteries only; Type V, combined features of Types IIb and IV. Angiographic classification was determined based on MRA findings obtained during routine clinical care.

### 2.6. Statistical Analysis

All statistical analyses were performed using IBM SPSS Statistics version 25.0 (IBM Corp., Armonk, NY, USA). Descriptive statistics were used to summarize demographic and clinical characteristics. Categorical variables were presented as frequencies and percentages, while continuous variables were expressed as mean ± standard deviation or median with interquartile range, as appropriate. Given the relatively small sample size, analyses were primarily descriptive. No formal statistical comparisons were performed to assess associations between angiographic classification and neurological involvement.

### 2.7. Ethics Statement

Ethics approval was obtained from the Clinical Research Ethics Committee of Istanbul University–Cerrahpaşa, Faculty of Medicine (approval date: 1 June 2021; document number: E-83045809-604.01.02-103273). The study was conducted in accordance with the principles of the Declaration of Helsinki. Informed consent was waived by the ethics committee given the retrospective nature of the study.

## 3. Results

### 3.1. Patient Characteristics

Among the 22 patients included in the study, 16 (73%) were female. The mean age at disease onset was 12.4 ± 5.3 years (range: 5–18 years). The median duration of follow-up for the entire cohort was 8.8 years (IQR, 7.1–12.5). Demographic, clinical, and neuroimaging characteristics of all patients are summarized in Table 1.

### 3.2. Neurological Clinical Features

Neurological involvement was identified in 5 of 22 patients (22.7%). Among these, three patients presented with ischemic stroke, whereas two were diagnosed with hypertensive encephalopathy.

Neurological symptoms were reported in 15 patients (68.2%). Headache was the most frequently observed symptom, occurring in 12 patients (54%). Among these, three patients (13.6%) were classified as having tension-type headache, while two (9%) exhibited a mixed headache pattern associated with both hypertension and tension-type features. Other reported neurological symptoms included transient paresthesia (*n* = 4, 18%), dizziness (*n* = 4, 18%), visual disturbances (*n* = 4, 18%), hemiparesis (*n* = 3, 13.6%), and syncope (*n* = 2, 9%).

Seizures were observed in three patients (13.6%) with neurological involvement. Two cases were attributed to hypertensive encephalopathy, while one occurred in the setting of acute ischemic stroke. Interictal sleep–wake EEG recordings were normal in all cases. These events were classified as acute symptomatic seizures, and long-term antiseizure therapy was not initiated.

On neurological examination, focal motor deficits were detected in three patients (13.6%), whereas tremor was observed in two patients (9%).

### 3.3. Neuroimaging Findings

**Figure 1:**
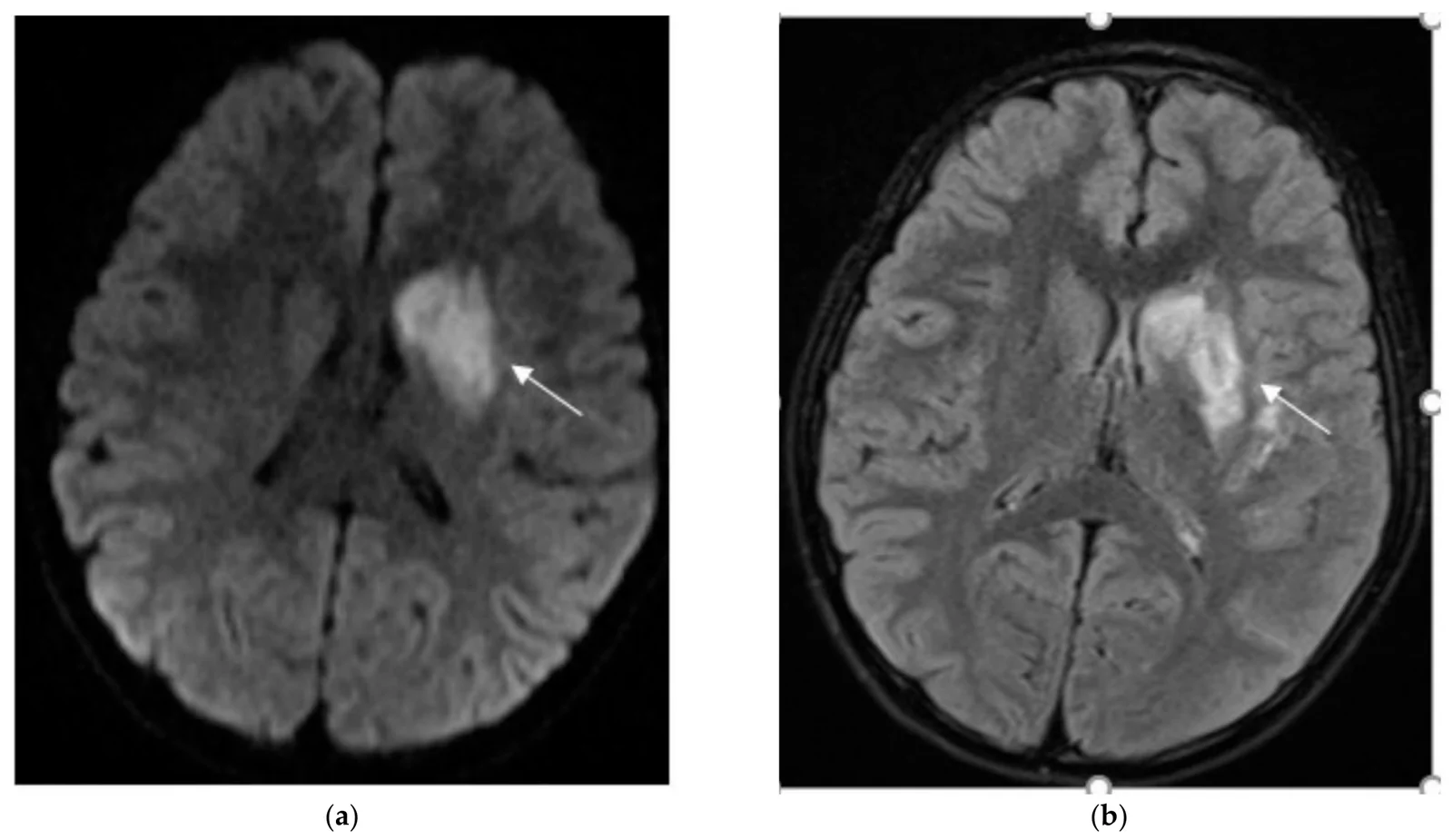

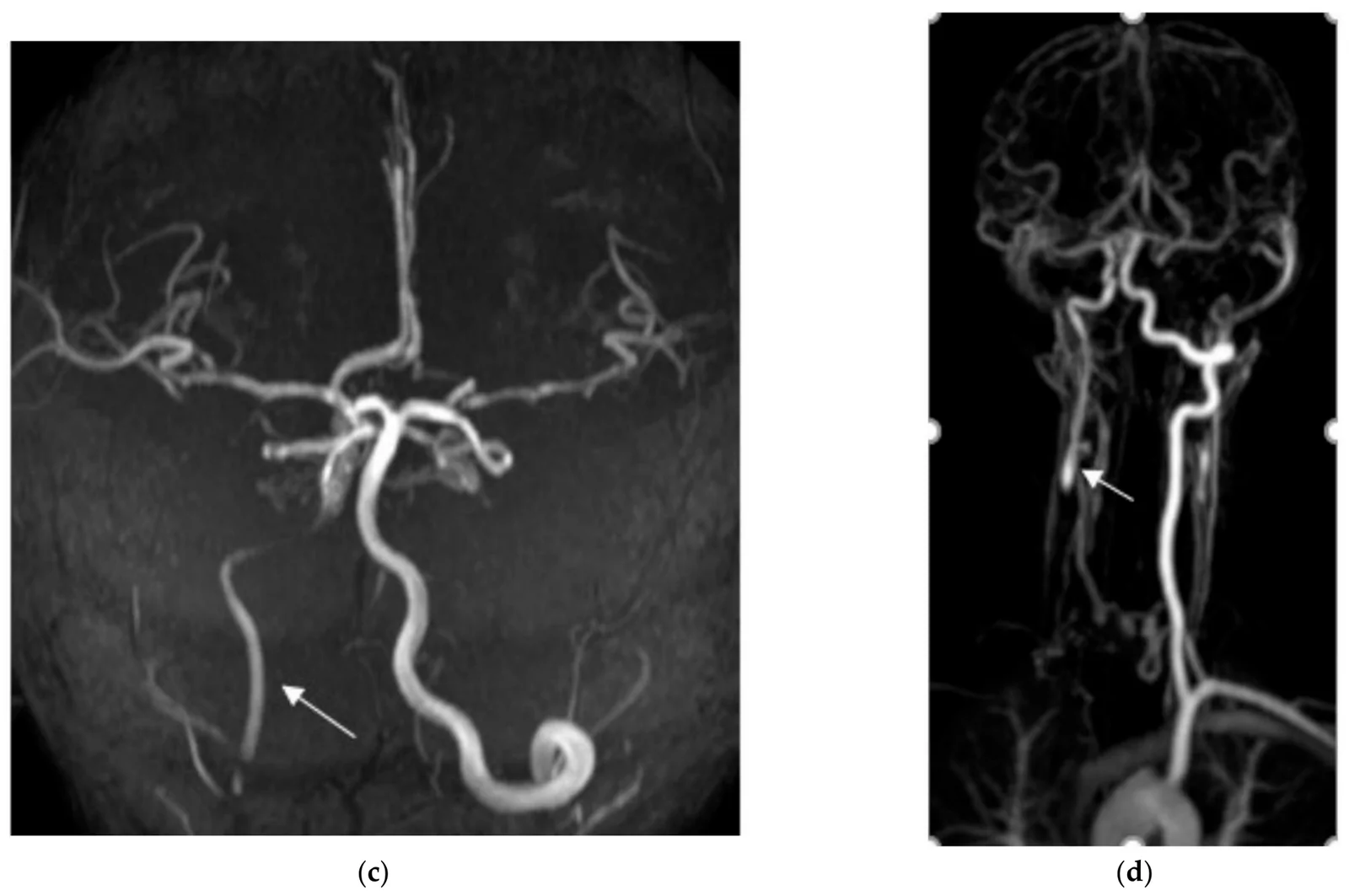
Neuroimaging findings in Case 1: a 13-year-old girl presenting with acute left hemisphere ischemia due to Takayasu arteritis. (**a**) Diffusion-weighted imaging (DWI) shows increased intensity in the left basal ganglia consistent with acute infarction. (**b**) Axial FLAIR image shows an area of cytotoxic edema resulting from an acute infarction in the left basal ganglia. (**c**) Intracranial magnetic resonance angiography (MRA) demonstrates collateral filling of the left middle cerebral artery (MCA) and anterior cerebral artery (ACA) through the circle of Willis. (**d**) Neck magnetic resonance angiography (MRA) demonstrates occlusion of the left common carotid artery from its origin. The right common carotid artery is filled via collateral circulation from the thyrocervical trunk of the left subclavian artery. The aortic arch gives rise only to the left subclavian artery. The right vertebral artery is partially filled via collateral circulation.

**Figure 2:**
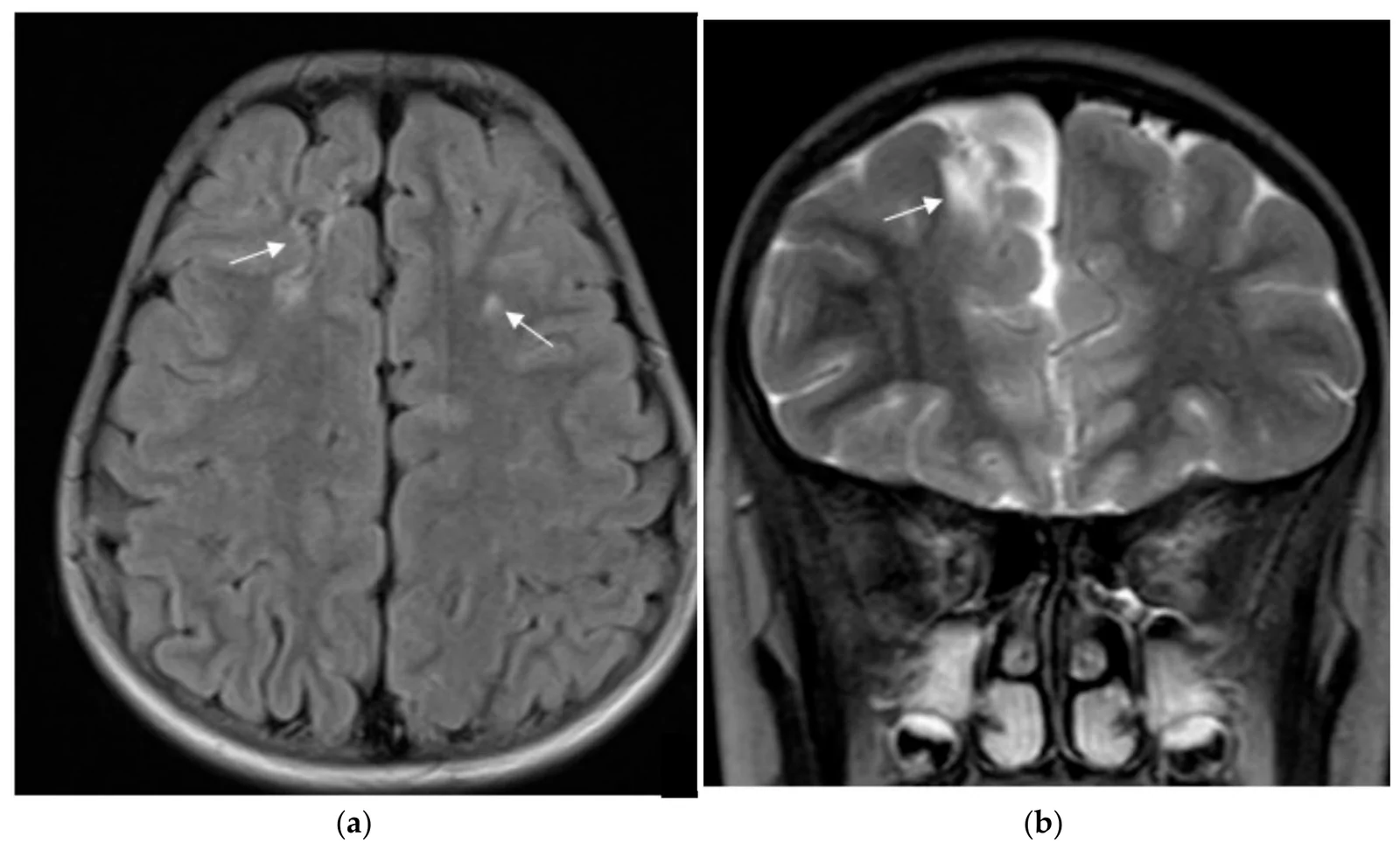

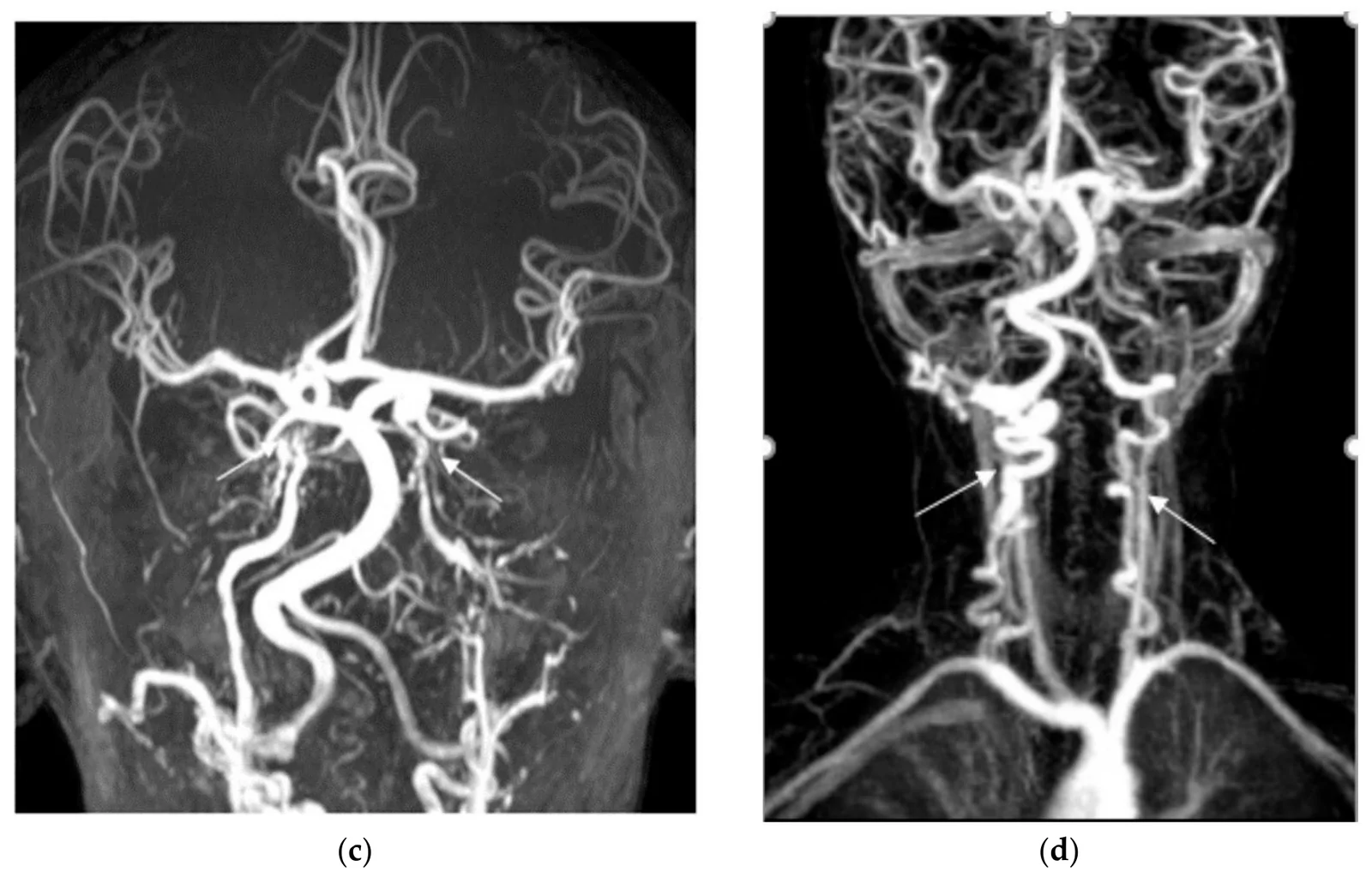
Neuroimaging findings in Case 2: an 8-year-old girl with chronic bilateral watershed infarctions due to bilateral internal carotid artery occlusion in Takayasu arteritis. (**a**) Axial FLAIR image demonstrates chronic watershed infarcts with encephalomalacia and surrounding gliosis, more prominent in the right hemisphere, with similar gliotic changes in the contralateral hemisphere. (**b**) Coronal T2-weighted image demonstrates chronic watershed infarction with encephalomalacia in the right hemisphere. (**c**) Intracranial magnetic resonance angiography (MRA) demonstrates distal occlusion of the bilateral internal carotid arteries with collateral supply from the posterior circulation. (**d**) Neck magnetic resonance angiography (MRA) demonstrates tortuosity of the bilateral common carotid and internal carotid arteries with distal occlusion of the bilateral internal carotid arteries.

**Figure 3:**
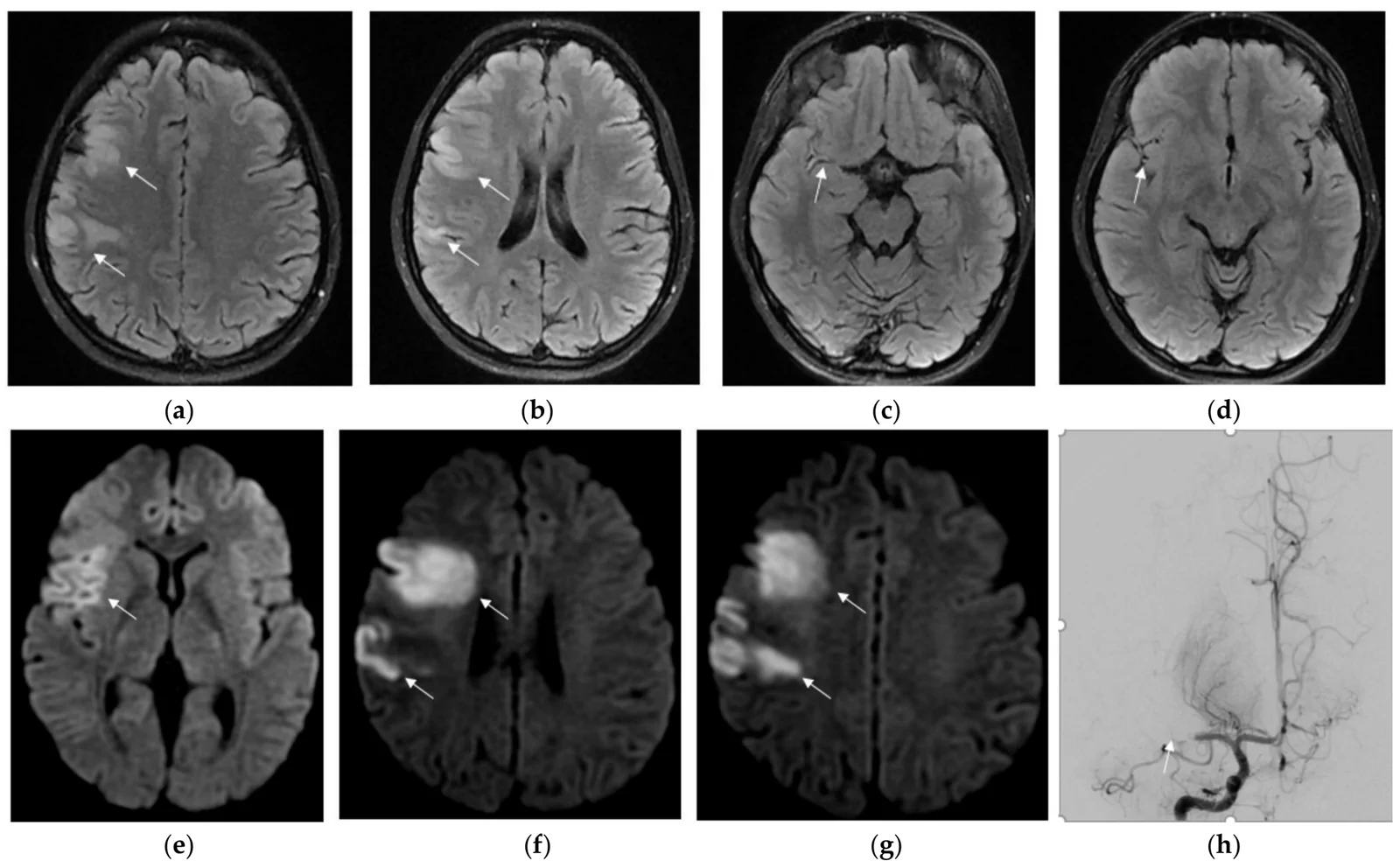
Neuroimaging findings in Case 3: a 16-year-old adolescent presenting with acute right MCA territory infarction in Takayasu arteritis. (**a**–**d**) Axial FLAIR images demonstrate hyperintense signal abnormalities in the right middle cerebral artery (MCA) territory consistent with acute ischemia. (**e**–**g**) Diffusion-weighted images demonstrate marked diffusion restriction in the right middle cerebral artery (MCA) territory consistent with acute infarction. (**h**) Digital subtraction angiography demonstrates distal occlusion of the right middle cerebral artery.

Cranial MRI was performed in all 22 patients. Abnormal findings were detected in five patients: cerebral ischemia or infarction in three patients, illustrated in the case presentations (Figure 1, Figure 2 and Figure 3), and nonspecific white matter signal changes in two patients that could not be definitively attributed to Takayasu arteritis.

In patients with ischemic stroke, MRA demonstrated significant cerebrovascular pathology, including arterial occlusion and collateral vessel formation, as further described in the illustrative case presentations.

### 3.4. Angiographic Classification

According to the angiographic classification system proposed by Numano et al., patients were distributed as follows: Type I in 2 patients, Type IIa in 1 patient, Type IIb in 3 patients, Type III in 3 patients, Type IV in 6 patients, and Type V in 7 patients. In one patient, the angiographic pattern progressed from Type I at initial presentation to Type V during follow-up, reflecting progressive and widespread vascular involvement.

### 3.5. Illustrative Cases of Cerebrovascular Involvement

Given the heterogeneity of neurological manifestations and patterns of vascular involvement, selected cases are presented to illustrate representative clinical and neuroimaging features within this cohort.

#### 3.5.1. Case 1

A 13-year-old girl presented with acute right-sided weakness, facial deviation, and speech disturbance, with examination confirming right hemiparesis involving the face. Cranial MRI demonstrated acute ischemia in the left basal ganglia (Figure 1a,b), and MRA showed collateral filling of the left MCA and ACA via the circle of Willis (Figure 1c), with occlusion of the left common carotid artery and collateralization from the left subclavian thyrocervical trunk (Figure 1d); Doppler ultrasonography confirmed thrombus formation in the right subclavian and axillary arteries. The patient improved with anticoagulation/antiplatelet therapy and immunosuppressive treatment (corticosteroids, azathioprine, cyclophosphamide, later tocilizumab), remaining stable over 9 years of follow-up with residual mild facial asymmetry and hemiparesis.

#### 3.5.2. Case 2

An 8-year-old girl presented with acute left-eye vision loss. She had a history of gait disturbance and recurrent headaches at age 6, when cranial MRI had already shown bilateral watershed T2/FLAIR hyperintensities, though this had not been further evaluated at the time. Current work-up revealed hypertension and elevated acute-phase reactants. MRI confirmed chronic bilateral watershed encephalomalacia with gliosis (Figure 2a,b), and MRA demonstrated distal bilateral internal carotid artery occlusion with posterior-circulation collateralization and a tortuous, dilated vertebrobasilar system (Figure 2c,d). She was treated with pulse corticosteroids, aspirin, and maintenance tocilizumab, remaining free of recurrent cerebrovascular events over 12 years of follow-up, with only mild residual motor apraxia.

#### 3.5.3. Case 3

A 16-year-old adolescent presented with headache, hypertension, an absent right radial pulse, and elevated acute-phase reactants. Vascular imaging showed an aberrant, severely stenotic (~90%) right subclavian artery with an interposed 12-mm aneurysmal segment and diffuse thoracoabdominal aortic wall thickening. During follow-up, the patient developed acute left-sided hemiparesis, speech disturbance, and seizures; FLAIR and diffusion-weighted MRI confirmed acute right MCA-territory infarction (Figure 3a–g), and MRA showed distal right MCA occlusion (Figure 3h), managed with endovascular thrombectomy and carotid angioplasty. Immunosuppressive therapy (high-dose corticosteroids, azathioprine, cyclophosphamide, later tocilizumab) was initiated, and mild residual left-sided facial hemiparesis persisted at last examination.

## 4. Discussion

Neurological manifestations in adult patients with Takayasu arteritis are well documented, with a reported incidence ranging from 57% to 80%, including dizziness, headache, visual disturbances or vision loss, stroke, and transient ischemic attack. However, despite being the most common large-vessel vasculitis in childhood, data on pediatric Takayasu arteritis remain limited. In particular, central nervous system (CNS) manifestations have been insufficiently explored in pediatric populations, especially from a pediatric neurology perspective [14,18]. In adults, ischemic events are more frequently attributed to atherosclerotic acceleration superimposed on vasculitic stenosis given the older age and longer disease duration at presentation, whereas in children, stroke more often reflects acute hemodynamic compromise from rapidly progressive vasculitis in the absence of significant atherosclerotic burden, which may explain differences in stroke timing and mechanism between the two populations [14,18].

In our cohort, neurological involvement was identified in 22.7% of patients, which was lower than some previously reported rates. For example, Eleftheriou et al. reported neurological involvement in 36% of children with Takayasu arteritis in a tertiary referral center. This discrepancy may be explained by our stricter definition of neurological involvement, which distinguished nonspecific neurological symptoms from objective central nervous system involvement based on clinical and/or neuroimaging findings [19]. Distinguishing objective CNS involvement from nonspecific neurological symptoms is clinically important because these two categories carry different prognostic and management implications: objective involvement (stroke, hypertensive encephalopathy) reflects irreversible or acute end-organ injury requiring urgent intervention and long-term surveillance, whereas nonspecific symptoms more often track with disease activity and blood pressure control and may resolve with systemic treatment alone. Applying a uniform definition that conflates these categories risks overestimating clinically significant CNS disease and may obscure the true burden of neurological morbidity. We therefore propose that future PTA cohorts adopt an explicit, two-tier framework when reporting neurological outcomes, to allow more meaningful comparison across studies. In the present cohort, well-defined neurological involvement mainly comprised ischemic stroke and hypertensive encephalopathy. Stroke has been reported in pediatric Takayasu arteritis cohorts with rates ranging from approximately 6% to 24%; for example, stroke was reported in 6% of patients in a Chinese pediatric cohort, 12% in a Mexican pediatric-onset cohort, and approximately 24% in a Singapore childhood-onset series [16,20,21].

These figures are broadly consistent with a recent systematic review and meta-analysis of predominantly adult TA cohorts, which reported a pooled stroke/TIA prevalence of 10.7% and identified stroke as responsible for 21.2% of TA-related deaths, underscoring the clinical importance of cerebrovascular surveillance across the age spectrum in this disease [22].

Similarly, in our cohort, stroke was observed in 13.6% of patients and was associated with significant morbidity. Notably, in two patients, ischemic stroke represented the initial manifestation of the disease, indicating that serious neurological complications may precede the diagnosis of Takayasu arteritis. This observation is clinically significant, as stroke—although uncommon—may be the first presentation of the disease. Failure to consider vasculitis in the differential diagnosis may result in delayed diagnosis and subsequent complications due to delayed initiation of appropriate treatment [10]. In addition, stroke may also occur during follow-up, highlighting the need for continued clinical vigilance even after diagnosis.

Several mechanisms may underlie ischemic stroke in PTA. Chronic hemodynamic compromise from progressive stenosis of the carotid or vertebral arteries can produce watershed infarction through sustained hypoperfusion, as illustrated in Case 2, where bilateral watershed injury developed in the setting of long-standing bilateral internal carotid artery occlusion. In contrast, territorial infarcts such as the MCA-territory events observed in Cases 1 and 3 are more consistent with in situ thrombosis or artery-to-artery embolism at sites of severe stenosis, occasionally precipitated by superimposed thrombus, as was demonstrated on Doppler ultrasonography in Case 1. The distinction between large-vessel (extracranial) stenosis and intracranial involvement is clinically relevant, since the former may be amenable to revascularization or antithrombotic therapy, whereas isolated intracranial disease—as in Case 3, where distal MCA occlusion required endovascular thrombectomy—may carry a different risk profile and treatment approach. Robust collateral circulation, frequently observed in our cohort through the circle of Willis or leptomeningeal pathways, likely modulates infarct severity and may explain why some patients with severe angiographic stenosis remained asymptomatic. Finally, severe or poorly controlled hypertension, most often secondary to renal artery involvement, may independently contribute to cerebral injury through impaired autoregulation, as reflected in the two cases of hypertensive encephalopathy in our cohort.

The neuroimaging findings in our cohort highlight patterns that merit closer attention. Watershed infarction, as seen in Case 2, reflects chronic hemodynamic insufficiency rather than embolic occlusion and should prompt evaluation for proximal large-vessel stenosis. Collateral circulation, well demonstrated on MRA in Cases 1 and 2, is an important marker of hemodynamic compensation and may help stratify stroke risk. Because bilateral distal internal carotid artery occlusion with basal collateral formation can radiologically resemble moyamoya syndrome, careful correlation with systemic inflammatory markers and extracranial vessel involvement is essential to avoid misdiagnosis in PTA; notably, a multi-contrast vessel wall MRI protocol combined with luminal imaging has been shown to differentiate moyamoya disease from atherosclerotic- and vasculitic-moyamoya syndromes substantially better than luminal imaging alone [23]. Emerging techniques such as vessel wall MRI, which can directly visualize arterial wall inflammation and distinguish active vasculitis from chronic fibrotic stenosis, and perfusion imaging, which may identify hemodynamically compromised but not-yet-infarcted territories, represent promising tools for future prospective studies in this population [23]. 

Another neurological complication observed in our cohort was hypertensive encephalopathy, which is an uncommon but well-recognized condition that typically occurs in the setting of severe or uncontrolled hypertension, most often secondary to renal artery involvement. Although hypertension is a common presenting feature of Takayasu arteritis, hypertensive encephalopathy has been reported only rarely [21]. Its exact prevalence is not well defined; however, it is considered to represent a small proportion of neurological manifestations reported in both pediatric and adult series.

Previous reports from pediatric cohorts have shown that the majority of neurological manifestations in pediatric Takayasu arteritis are nonspecific and are more frequently associated with systemic disease activity and hypertension rather than direct parenchymal CNS involvement. In a large Brazilian multicenter study including 71 pediatric patients, neurological symptoms were reported in approximately 70% of cases at disease onset, with the majority being nonspecific in nature [24]. Consistent with these observations, neurological symptoms were present in 68.2% of patients in our cohort and were predominantly nonspecific, closely associated with hypertension and systemic inflammation rather than direct CNS vasculitis. Symptoms such as headache, dizziness, and syncope are commonly observed, whereas overt cerebrovascular events are less frequent. These findings have been supported by multicenter pediatric studies, which emphasize that neurological complaints in pediatric Takayasu arteritis often reflect hemodynamic disturbances or inflammatory burden rather than structural brain injury [9,25]. 

Headache was the most frequently reported neurological symptom in our cohort, occurring in 54% of patients, which appears higher than previously reported pediatric cohorts, where headache has been reported in approximately 27–40% of children, although higher rates have also been described in some series [9,14,25]. 

The relatively higher frequency of headache in our cohort may be explained by the routine neurological follow-up and detailed assessment of headache symptoms during clinical evaluation. Although headache was most commonly attributed to hypertension, three patients fulfilled the diagnostic criteria for tension-type headache and were referred for psychological evaluation and management.

The frequency of syncope varies from 4.2% to 18.8% and seizures are reported in 6.3–27.2% of children with Takayasu arteritis [14]. All seizures in our cohort were classified as acute symptomatic events and did not recur during follow-up. The nonspecific symptoms observed—including syncope, dizziness, paresthesia, and visual disturbances—are attributed to cerebral hypoperfusion secondary to arterial involvement and are of considerable importance for early diagnosis.

Angiographic subtypes involving the aortic arch and its branches—particularly Numano Type I and Type IIb—have been associated with an increased risk of ischemic CNS complications [8,12]. Previous studies have reported a higher incidence of cerebral infarcts in patients with predominant upper aortic arch involvement [26]. However, recent large pediatric series indicate that childhood-onset Takayasu arteritis more commonly presents with extensive angiographic patterns. In a systematic review including 636 pediatric patients, Type V was the most prevalent subtype (53%), followed by Type IV (21%) [9]. Consistent with these findings, the most frequent angiographic subtypes in our cohort were Type V and Type IV, reflecting a predominance of diffuse vascular involvement. Importantly, neurological symptoms were also observed in patients with these subtypes, suggesting that mechanisms such as hypertension, vascular stiffness, and global hemodynamic alterations may contribute to neurological manifestations beyond direct carotid or vertebral artery involvement.

Neuroimaging findings in pediatric Takayasu arteritis remain heterogeneous, ranging from normal imaging to ischemic or vascular abnormalities. In our cohort, MRI revealed ischemic lesions in a subset of patients, as well as nonspecific white matter changes, highlighting the importance of neuroimaging in detecting both clinically overt and subclinical CNS involvement [14].

In the present cohort, PTA demonstrated a clear female predominance, with disease onset occurring mainly in late childhood and early adolescence, consistent with previously reported pediatric and adult series [27,28]. A major strength of this study is the detailed long-term follow-up of a rare disease cohort by a multidisciplinary team comprising rheumatology and neurology specialists. However, the study is limited by its retrospective design and single-center setting, which resulted in a relatively small sample size. In addition, standardized functional outcome measures such as the modified Rankin Scale or the Pediatric Stroke Outcome Measure were not systematically applied in this retrospective cohort; neurological outcomes were instead based on qualitative clinical examination findings. This limits the ability to precisely quantify long-term functional disability and should be addressed prospectively in future studies.

## 5. Conclusions

In conclusion, patients with pediatric Takayasu arteritis should be closely monitored through multidisciplinary follow-up involving both rheumatology and neurology clinics. Particular attention should be given to early and nonspecific neurological manifestations. In patients presenting with symptoms suggestive of cerebral hypoperfusion, vasculitides—including Takayasu arteritis—should be considered in the differential diagnosis. Systematic neurological evaluation and the use of neuroimaging modalities such as MRI and MRA play a crucial role in identifying early or subclinical CNS involvement and guiding clinical management.

## Figures and Tables

**Table 1:** Clinical characteristics, neurological symptoms, neuroimaging findings, and outcome of 22 patients with pediatric Takayasu arteritis.

Patient	Sex	Numano Type (Onset → Last)	Neurological Symptoms									MRI Findings	Final Neurological Examination
			Headache	Visual Dist.	Dizziness	Paresthesia	Tremor	Paresis	Stroke	Seizure	Syncope		
Total positive, n (%)			12 (55%)	4 (18%)	4 (18%)	4 (18%)	2 (9%)	3 (14%)	3 (14%)	3 (14%)	2 (9%)		
Patient 1	F	IIb → IIb	+	+	−	−	−	−	−	+	+	Right temporofrontal chronic infarct (post-coronary artery bypass surgery); retinal hemorrhages—neurological findings not solely attributable to TA	Normal
Patient 2	F	V → V	−	−	−	−	−	−	−	−	−	Normal	Normal
Patient 3	F	IIb → IIb	+	+	+	+	−	−	−	−	+	Normal	Normal
Patient 4	M	IV → IV	−	−	−	−	−	−	−	−	−	Normal	Normal
Patient 5	F	V → V	−	−	−	−	−	−	−	−	−	Normal	Normal
Patient 6	F	I → I	+	+	+	−	−	+	+	−	−	Acute infarction: left caudate nucleus and lentiform nucleus (DWI/FLAIR)	Right hemiparesis; mild left facial palsy
Patient 7	F	IV → IV	−	−	−	−	−	−	−	−	−	Normal	Normal
Patient 8	M	IIa → IIa	−	−	−	−	−	−	−	−	−	Frontal gliotic focus (clinically insignificant)	Normal
Patient 9	F	V → V	+	−	+	+	+	+	+	+	−	Acute infarction: right MCA territory (DWI/FLAIR); MCA occlusion on MRA	Left arm paresis
Patient 10	F	IV → IV	−	−	−	−	−	−	−	−	−	Normal	Normal
Patient 11	F	III → III	+	−	−	+	−	−	−	−	−	Normal	Normal
Patient 12	F	III → III	−	−	−	−	−	−	−	−	−	Normal	Normal
Patient 13	M	V → V	−	−	−	−	−	−	−	−	−	Normal	Normal
Patient 14	M	IV → IV	+	−	−	−	−	−	−	−	−	Normal	Normal
Patient 15	F	IIb → IIb	+	−	+	−	−	−	−	−	−	Normal	Normal
Patient 16	F	I → V	+	+	−	−	−	+	+	−	−	Vertebrobasilar fusiform changes; frontal T2/FLAIR hyperintensity	Normal
Patient 17	F	V → V	−	−	−	+	−	−	−	−	−	Normal	Normal
Patient 18	F	IV → IV	−	−	−	−	−	−	−	+	−	Bilateral frontal and left parietal subcortical T2/FLAIR hyperintense foci; cerebral atrophy	Normal
Patient 19	F	V → V	+	−	−	−	+	−	−	−	−	Normal	Normal
Patient 20	M	IV → IV	+	−	−	−	−	−	−	−	−	Normal	Normal
Patient 21	M	III → III	+	−	−	−	−	−	−	−	−	Normal	Normal
Patient 22	F	V → V	+	−	−	−	−	−	−	−	−	Normal	Normal

+, present; −, absent; dist., disturbance; DWI, diffusion-weighted imaging; FLAIR, fluid-attenuated inversion recovery; MCA, middle cerebral artery; MRA, magnetic resonance angiography; TA, Takayasu arteritis. Rows highlighted in yellow indicate patients with ischemic stroke or acute symptomatic seizure. Patient 16 showed angiographic progression from Numano Type I to Type V during follow-up.

## Data Availability

The data presented in this study are available on reasonable request from the corresponding author. The data are not publicly available due to privacy restrictions.
